# Effect of YC-1102 on the Improvement of Obesity in High-Fat Diet-Induced Obese Mice

**DOI:** 10.3390/cimb46020093

**Published:** 2024-02-07

**Authors:** Hwa-Young Yu, Kyoung Kon Kim, Sin Hwa Baek, Cho I Park, Hye Jin Jeon, Ae Ri Song, Hyun-Je Park, Il Bum Park, Jong Soo Kang, Jung Min Kim, Tae Woo Kim, Sun Min Jang, Joo Young Cha, Junghyun Kim

**Affiliations:** 1 Department of Oral Pathology, School of Dentistry, Jeonbuk National University, Jeonju 54907, Republic of Korea; naive17jbnu@gmail.com; 2 Newgen Healthcare Co., Ltd., 56 Soyanggang-ro, Chuncheon-si 24232, Republic of Korea; gon87@newgenhc.co.kr (K.K.K.); jmkim@newgenhc.co.kr (J.M.K.); topclass@newgenhc.co.kr (T.W.K.); sunmin@newgenhc.co.kr (S.M.J.); 3 Yuhan Care Co., Ltd., Yuhan Care R&D Center, Yongin-si 17084, Republic of Korea; shbaek@yuhancare.com (S.H.B.); cipark@yuhancare.com (C.I.P.); hjjeon@yuhancare.com (H.J.J.); aeri.song@yuhancare.com (A.R.S.); hjpark@yuhancare.com (H.-J.P.); joshua.park@yuhancare.com (I.B.P.); 4 Yuhan Care Co., Ltd., Yuhan Natural Product R&D Center, Andong-si 36618, Republic of Korea; 5 Yuhan Care Co., Ltd., Seoul 07335, Republic of Korea; jskang@yuhancare.com; 6 Department of Molecular Medicine and Biopharmaceutical Sciences, Graduate School of Convergence Science and Technology, Seoul National University, Seoul 08826, Republic of Korea; 7 Non-Clinical Evaluation Center Biomedical Research Institute, Jeonbuk National University Hospital, Jeonju 57907, Republic of Korea

**Keywords:** obesity, adipogenesis, high-fat diet, *Rosa multiflora*, YC-1102

## Abstract

Obesity is one of the major risk factors for metabolic diseases worldwide. This study examined the effects of YC-1102, an extract derived from the roots of *Rosa multiflora*, on 3T3-L1 preadipocytes and high-fat diet (HFD)-induced obese mice. In vivo experiments involved the oral administration of YC-1102 (100, 150, and 200 mg/kg body weight) daily to mice for eight weeks. YC-1102 was found to downregulate the expressions of PPARγ and C/EBPα during adipogenesis, inhibiting adipocyte differentiation and upregulating the expression of PGC-1α for energy metabolism to enhance mitochondrial biogenesis and fatty acid oxidation. It has been shown that daily administration of YC-1102 to mice receiving a HFD prevented an increase in body weight and the accumulation of body fat. YC-1102 administration also reduced TG, TC, and LDL cholesterol levels, as well as glucose and leptin levels, and increased adiponectin levels, thus effectively inhibiting the metabolism of lipids. YC-1102-treated mice showed significant reductions in the mRNA expression of *PPARγ* and *C/EBPα*. The levels of *PGC-1α* involved in energy metabolism increased significantly in the YC-1102-treated mice when compared to the HFD-treated mice. According to the findings of this study, YC-1102 has a dual mechanism that reduces transcription factors that promote the differentiation of adipocytes and increases transcription factors that promote energy consumption.

## 1. Introduction

In the world, obesity is considered to be a major public health concern as a result of excessive body fat that results from a chronic energy imbalance caused by excessive dietary intake and insufficient physical activity [1]. Obesity is associated with various metabolic diseases, including type II diabetes mellitus, dyslipidemia, fatty liver disease, hypertension, and cancer. Obesity can be treated through lifestyle modification, pharmacotherapy, and bariatric surgery. Currently, researchers are investigating improved nutritional and nutraceutical products for obesity patients. An imbalance between energy expenditure and absorption results in the growth of adipose tissue, resulting in obesity [2]. Some of the complications associated with obesity include non-alcoholic fatty liver disease and non-alcoholic steatohepatitis, chronic inflammation, and risk of type II diabetes and cardiovascular disease [3,4,5]. Due to the coronavirus disease 2019 (COVID-19) pandemic, lifestyle and dietary habits, as well as decreased physical activity levels, have contributed to the rise in obesity rates [6,7].

In response to a high-fat diet (HFD), mature adipocytes undergo hypertrophy or hyperplasia that allows triglycerides (TGs) to be stored in white adipose tissue (WAT) [2,3]. In the process of differentiation of adipocytes, progenitors undergo mitogenic expansion and acquire mature characteristics. WATs are classified into two types: subcutaneous and visceral, and their enlargement contributes to the development of obesity and metabolic complications [6]. There has been evidence that transcription factors involved in adipogenesis include peroxisome proliferator-activated receptors (PPARs), CCAAT/enhancer-binding protein alpha (C/EBPα), and sterol regulatory element-binding protein-1c (SREBP-1c), which all activate genes downstream involved in adipogenesis and lipid accumulation [8,9]. An increase in the expression of transcription factors and lipogenic enzymes is observed in 3T3-L1 preadipocytes, resulting in their differentiation into adipocytes. There are several numbers of key lipogenic enzymes that play a vital role in lipid synthesis including fatty acid synthase, acetyl CoA carboxylase, and stearoyl desaturase-1 [3]. Energy metabolism occurs in adipose tissue through the activation of sirtuin-1, PR domain-containing-16, and PPARγ coactivator (PGC)-1α mediated by uncoupling protein (UCP)-1 [9,10,11,12,13]. To manage obesity, adipogenesis control and energy metabolism activation in adipose tissue might be useful [14,15].

A few drugs have been ©d for treating obesity; however, their limitations remain due to several adverse effects. As an example, orlistat inhibits the digestion of lipids by binding covalently to lipase active sites, but it is associated with side effects such as steatorrhea and abdominal gas [16]. As another drug for the treatment of obesity, sibutramine reduces food intake and improves satiety, but it has been associated with the development of cardiovascular diseases [17]. Consequently, it is necessary to develop a new strategy for treating obesity in a safe and effective manner. Recently, functional foods, such as natural plant products, have attracted increasing attention due to their anti-obesity properties and long-term safety.

The Rosa multiflora plant is a member of the Rosaceae family. The roots of this plant contain tormentic acid, a triterpenoid, rosamultin, and condensed tannin compounds, including procyanidin B3 and catechin, which are known to be powerful antioxidants [18,19]. It was found that rosamultin reduced triglyceride levels in 3T3-L1 cells [20] and inhibited hepatic lipid peroxidation that was induced by promobenzene in rats [21]. Previous studies have shown that *R. multiflora* significantly lowers blood lipid levels in laboratory animals and exhibits anti-inflammatory and antioxidant properties [22,23]. There is, however, a lack of research on the anti-obesity properties of *R. multiflora*. The purpose of this study was to determine the effect of an extract of *R. multiflora* roots (YC-1102, Rosa Flora^TM^) on pre-adipose cell differentiation in 3T3-L1 cells and a HFD-fed mouse model.

## 2. Materials and Methods

### 2.1. YC-1102 Preparation and Standardization

Standardized YC-1102 (production code name: KWFD-H01-T5) was provided by YuhanCare Co., Ltd. (Gyeonggi, Republic of Korea). YC-1102 was extracted from the roots of *R. multiflora*. *R. multiflora* was purchased from the Goesan Herbal market (Goesan, Republic of Korea). Voucher specimens were deposited in the herbal bank at the Biological Resource Center, Korea University (voucher number: EKORPL201908090141). The extraction was performed at 60 °C for 5 h using 70% ethanol at 10.7 (*w*/*v*) based on the dry weight of *R. multiflora* roots. The extract was concentrated using a 1 μm filter. The enriched YC-1102 was spray-dried using a spray drier (MH-8, Mehyun Engineering Ltd., Anyang-City, Gyeonggi-do, Republic of Korea) at an inlet temperature of 170 ± 5 °C and an outlet temperature of 100 ± 5 °C. As part of the validation of the YC-1102 solution, liquid chromatography-mass spectrometry (LC-MS/MS) analysis was performed on active compounds. LC-MS/MS system consisted of an Ultimate 3000 UHPLC (Thermo Scientific, Waltham, MA, USA) coupled to a Triple TOF 5600+ (AB Sciex, Forster City, CA, USA) fitted with an electrospray ionization source. The YC-1102 solution was standardized via high-performance liquid chromatography (HPLC) analysis of rosamultin content. The ACQUITY UPLC H class (Waters, Milford, MA, USA), which was used to conduct the HPLC analysis, included a quaternary pump (ACQUITY QSM) and a UV/vis detector (ACQUITY UPLC TUV) that can detect an output signal at a wavelength of 205 nm. The HPLC analysis was repeated three times at different time points to measure the rosamultin ©ontent in YC-1102.

### 2.2. Cell Viability Assay

3T3-L1 pre-adipocytes were purchased from ATCC (American Type Culture Collection, Manassas, VA, USA) and cultured with Dulbecco’s modified Eagle’s medium (DMEM, Welgene, Daegu, Republic of Korea) containing 10% fetal bovine serum (FBS, Welgene) and 1% penicillin–streptomycin (Welgne) at 37 °C and 5% CO_2_. The methylthialazole tetrazolium (MTT) assay was performed to evaluate the cytotoxicity of YC-1102. For this, 100 μL of cells was aliquoted into each well of a 96-well plate at 1 × 10^5^ cells/mL and cultured for 24 h. Thereafter, cells were treated with serum-free medium containing varying concentrations of diluted YC-1102. After another 24 h, the culture medium containing YC-1102 was removed, and 100 μL of culture medium with dissolved MTT (0.5 mg/mL, Sigma-Aldrich, St. Louis, MO, USA) was aliquoted for 4 h culture at 37 °C. Next, the culture medium was removed, and the formazan formed in each well was dissolved in 100 μL of DMSO; then, the absorbance was measured at 570 nm after 30 min using a UV/vis spectrophotometer (Optizen 2120UV plus, Mecasys Co., Ltd., Daejeon, Republic of Korea). The cytotoxicity was determined based on the reference absorbance of the control group.

### 2.3. Triglyceride Assay

The cells were aliquoted into a 6-well plate at 5 × 10^5^ cells/well and cultured to full density to induce the differentiation of 3T3-L1 preadipocytes into adipocytes. After two days of culture, the cells were then cultured with DMEM containing 10% FBS and a mixture of 0.5 mM 3-isobutyl-1-metylxanthine (IBMX), 0.5 μM dexamethasone, and 10 μg/mL insulin solution for another two days for the onset of differentiation. The medium was then replaced with DMEM containing 10 μg/mL insulin and 10% FBS for two days of differentiation. The cells were subsequently cultured in DMEM containing only 10% FBS for four days to complete adipocyte differentiation, which was marked by the formation of lipid droplets due to lipid accumulation in the cells. To measure the intracellular triglyceride content, the cells were treated with YC-1102 (at 50, 100, or 200 μg/mL) with a supply of fresh medium and MDI solution, to be cultured until the end of the differentiation induction. Afterward, the differentiated cells were washed twice with PBS, homogenized using NP40, and heated to 100 °C to dissolve the intracellular TG content. An EZ-Triglyceride Quantification Assay Kit (Dogenbio, Seoul, Republic of Korea) was used to measure TG content following the manufacturer’s protocols.

### 2.4. Real-Time PCR

Total RNA was extracted from epididymal white adipose tissue (eWAT) with TRI reagent (Invitrogen, Carlsbad, CA, USA), and cDNA was generated with PrimeScript Reverse Transcriptase kit (Takara Bio, Kusatsu, Japan) according to the manufacturer’s instructions. Using a SYBR Green PCR Master Mix (Thermo Fisher Scientific, Waltham, MA, USA) and an ABI 7500 Real-Time PCR System (Applied Biosystems, Foster City, CA, USA), cDNA was synthesized and subjected to real-time PCR analysis. Gene expression analysis involved the relative quantification method with GAPDH normalization, followed by the calculation of fold change. The primers are listed in Table 1.

### 2.5. Western Blot Analysis

The cells were lysed with RIPA buffer and quantified using the Bradford method. Then, 20 μg of proteins was electrophoresed through SDS-PAGE and transferred to a polyvinylidene fluoride membrane (0.45 μm, Thermo Fisher Scientific). After blocking, the membrane was treated first with the primary antibody (C/EBPα (1:1000, Cell Signaling Technology, Danvers, MA, USA), PPARγ (1:1000, Cell Signaling Technology), PGC-1α (1:1000, Santa Cruz Biotechnology, Santa Cruz, CA, USA), UCP-1 (1:1000, Cell Signaling Technology)), and then with the secondary antibody (horseradish peroxidase-conjugated secondary antibody (1:1000, Cell Signaling Technology). Protein expression levels were determined using an image analyzer (LAS4000, Fujifilm, Tokyo, Japan). The intensity of the detected bands was analyzed using ImageJ software (Version 1.53, NIH, Bethesda, MD, USA).

### 2.6. Animals and Experimental Design

Six-week-old male C57BL6/J mice were purchased from KoaTech (Pyeongtaek-si, Gyeonggi-do, Republic of Korea). All procedures for animal experiments complied with guidelines regarding animal management. The study protocol was approved by the Institutional Animal Care and Use Committee of Jeonbuk National University Hospital (JBNUH-IACUC-2021-42). The experimental groups included one normal diet group (ND) and six HFD (rodent diet with 60% kcal fat, D16042106, Research Diet, New Brunswick, NJ, USA)-fed groups. Rats were randomly divided into 7 groups of 7~8 rats as follows: (1) normal diet (ND)-fed mice (*n* = 7), (2) vehicle-treated HFD-fed mice (*n* = 8), (3) HFD-fed mice treated with YC-1102 (100 mg/kg body weight, *n* = 8), (4) HFD-fed mice treated with YC-1102 (150 mg/kg body weight, *n* = 8), (5) HFD-fed mice treated with YC-1102 (200 mg/kg body weight, *n* = 8), (6) HFD-fed mice treated with *Garcinia cambogia* (hydroxycitric acid, HCA, 400 mg/kg body weight, *n* = 8), and (7) HFD-fed mice treated with *Cissus quadrangularis* (Cissus, 65 mg/kg body weight, *n* = 8). The oral doses of YC-1102 were selected based on the preliminary animal study and the NOEAL (no-observed-adverse-effect level, 5000 mg/kg/day) in a 90-day repeated oral toxicity study. We also chose the doses of HCA and Cissus based on previous report [24] and human equivalent dose conversion. HCA and Cissus, provided by YuhanCare Co., Ltd. (Gyeonggi, Republic of Korea), are well-known herbal dietary supplements used to treat obesity and were used as comparators. YC-1102, HCA, and Cissus were dissolved in distilled water and administered once a day through oral gavage for eight weeks. Body weight and food intake were recorded weekly for eight weeks. At the end of the experimental period, body fat was measured using dual-energy X-ray absorptiometry (DEXA, Medikors, Seongnam, Republic of Korea). After overnight fasting, the mice were sacrificed to measure organ weights. Relative body weight (%) was calculated by the following equation. Relative weight (%) = body weight (g)/initial body weight (g) × 100.

### 2.7. Serological Assay

Using the serum reserved during the autopsy, the TG, total cholesterol (TC), high-density lipoprotein cholesterol (HDL-C), low-density lipoprotein cholesterol (LDL-C), and glucose levels were measured using the corresponding reagents (Asan Pharmaceutical, Seoul, Republic of Korea). Blood insulin (Biovision, Milpitas, CA, USA), leptin (Cusabio, Wuhan, China), and adiponectin (Cusabio) levels were measured according to the manufacturer’s instructions.

### 2.8. Histopathological Test

The eWAT isolated from the testes of the animals was fixed in 10% formalin. The adipose tissue was then embedded in paraffin and sliced. Next, the adipose tissues were stained with hematoxylin and eosin, and each stained tissue was observed and photographed using a light microscope (Olympus, Tokyo, Japan). Subsequently, the adipocytes of at least 1000 cells were measured using ImageJ software.

### 2.9. Statistical Analysis

GraphPad Prism 9.0.1 (GraphPad, San Diego, CA, USA) was used for statistical analysis. One-way ANOVA was performed following Levene’s test, and the significance of between-group variation was confirmed. A post hoc test was performed depending on the homogeneity of dispersion (Tukey’s multiple comparisons test in the case of homogeneous dispersion or Dunnett’s T3 test in the case of heterogeneous dispersion). All data were expressed as means ± standard deviation (SD).

## 3. Results

### 3.1. Identification of Rosamultin and Standardization of YC-1102 via LC-MS/MS and HPLC

**Figure 1:**
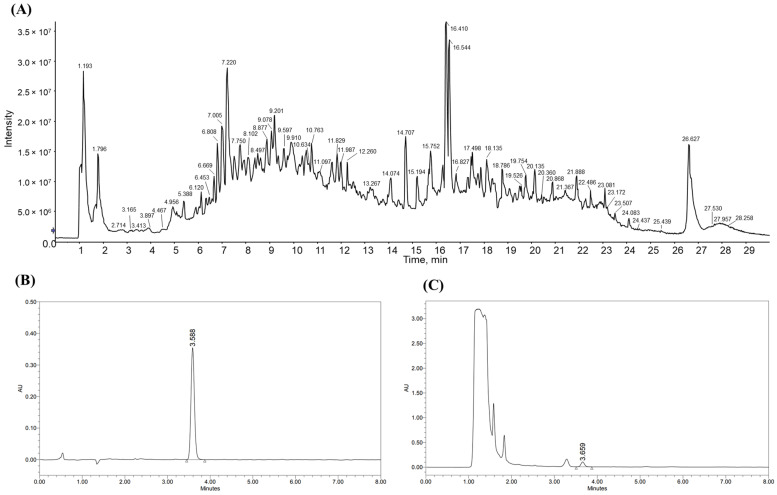
(**A**) Liquid chromatography-tandem mass spectrometry at 30–2000 mz for YC-1102. High-performance liquid chromatography (HPLC) profiles of (**B**) standard of rosamultin and (**C**) YC-1102.

The analysis of rosamultin content for standardization was performed with HPLC, and the LC-MS/MS analysis of YC-1102 showed that it contained various biologically active compounds (Figure 1A). The molecular formula and weight of the index compound, rosamultin, are C_36_H_58_O_10_ and 650.8 g/mol. The rosamultin content of the YC-1102 was measured using HPLC for standardization. The analysis confirmed that the retention time of rosamultin was 3.659 min and the content was 13.12 mg/g (Figure 1B,C). YC-1002 also contained several minor components, such as euscaphic acid and β-sitosterol.

### 3.2. YC-1102 Regulates Adipogenesis and Energy Metabolism in 3T3-L1 Cells

**Figure 2:**
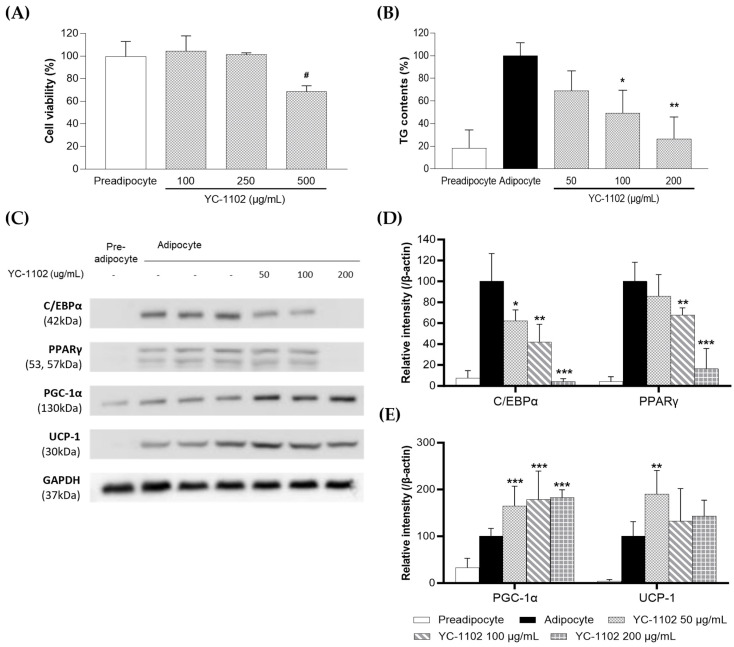
Effects of YC-1102 on adipogenesis in 3T3-L1 adipocytes. Cells were differentiated into adipocytes in the presence or absence of YC-1102. (**A**) Cell viability assay of YC-1102 in 3T3-L1 preadipocyte. (**B**) Quantified contents of intracellular triglyceride accumulation. (**C**–**E**) A Western blot analysis was performed to determine the expression levels of PPARγ, C/EBPα, PGC-1α, and UCP-1. Values are expressed as the mean ± SD. Each data point represents the mean value of samples in triplicate (WB) or quadruplicate (triglyceride assay and qPCR). ^#^ *p* < 0.05 vs. preadipocyte, * *p* < 0.05, ** *p* < 0.01, and *** *p* < 0.001 vs. adipocyte group.

To determine the cytotoxicity of YC-1102, we conducted cell viability assays using 3T3-L1 pre-adipocytes. The results indicate that YC-1102 was safe up to a concentration of 250 μg/mL (Figure 2A). Therefore, in this study, we determined the effect of YC-1102 on 3T3-L1 cells at non-toxic concentrations (50, 100, and 200 μg/mL). When pre-adipocytes differentiate into adipocytes, intracellular lipid droplets accumulate. Here, the induction of differentiation increased the TG content in 3T3-L1 cells; however, the TG content significantly decreased upon YC-1102 treatment in a concentration-dependent manner (Figure 2B). During the adipocyte differentiation, cell viability was not affected by YC-1102. To determine the effects of YC-1102 on adipogenesis and energy metabolism, the expressions of PPARγ, C/EBPα, PGC-1α, and UCP-1 were examined in differentiated 3T3-L1 cells. YC-1102 inhibited the expressions of PPARγ and C/EBPα, which regulate adipogenesis in 3T3-L1 adipocytes (Figure 2C,D). In contrast, the expression of PGC-1α, a protein that regulates energy metabolism, was significantly increased (Figure 2C,E). These results suggest that YC-1102 can help reduce body fat by inhibiting adipogenesis via the inhibition of PPARγ and C/EBPα and promote energy metabolism by increasing the expression of PGC-1α.

### 3.3. YC-1102 Reduces the Body Weight and Body Fat of HFD-Induced Obese Mice

**Figure 3:**
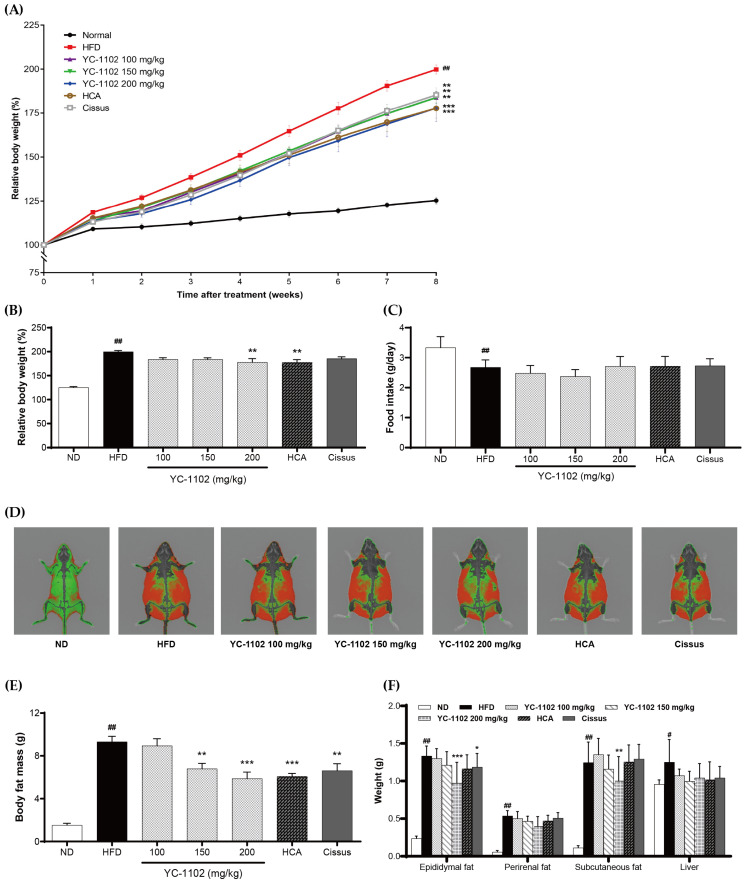
Effect of YC-1102, HCA, and Cissus on body weight (**A**), relative body weight at 8 weeks (**B**), average feed intake (**C**), body composition analysis (using DEXA analyzer) (**D**), body fat mass (**E**), and white adipose tissue weights (**F**) in HFD-fed mice. Values are expressed as the mean ± SD (ND group: n = 7 mice/group; other groups: n = 8 mice/group). # *p* < 0.05 and ## *p* < 0.001 vs. ND group, * *p* < 0.05, ** *p* < 0.01, and *** *p* < 0.001 vs. HFD group.

Weight changes were observed at 1-week intervals to determine the effect of YC-1102 administration on weight gain in obese mice fed an HFD for 8 weeks. The weight of mice in the HFD group was significantly higher than that in the ND group (Figure 3A). At week 8, a significant loss of weight was observed in the YC-1102 200 mg/kg group, with an average weight loss of approximately 22% lower than that in the HFD group; however, there was no difference in dietary intake between the groups (Figure 3B,C). Figure 3D,E show the comparisons of body fat mass between groups using DEXA. The results show that, compared to the ND group, the HFD group accumulated significantly more fat (red color) in the body cavity and subcutaneous tissue. Meanwhile, a significant reduction in body fat was observed in the YC-1102 diet groups when compared with the positive control groups, i.e., HCA and Cissus (Figure 3D,E). The measured weights of the epididymal fat, perirenal fat, subcutaneous fat, and liver of the test animals indicated that the weights were markedly higher in the HFD than in the ND group. In the YC-1102 groups, the organ weights that were increased by the HFD tended to decrease, particularly for epididymal fat and subcutaneous fat in the high-dose YC-1102-treated group (Figure 3F). Based on this result, the administration of YC-1102 was shown to effectively ameliorate the weight gain caused by a HFD as a result of reduced body fat mass.

### 3.4. Effects of YC-1102 on Biochemical Parameters in the Serum of HFD-Induced Obese Mice

**Figure 4:**
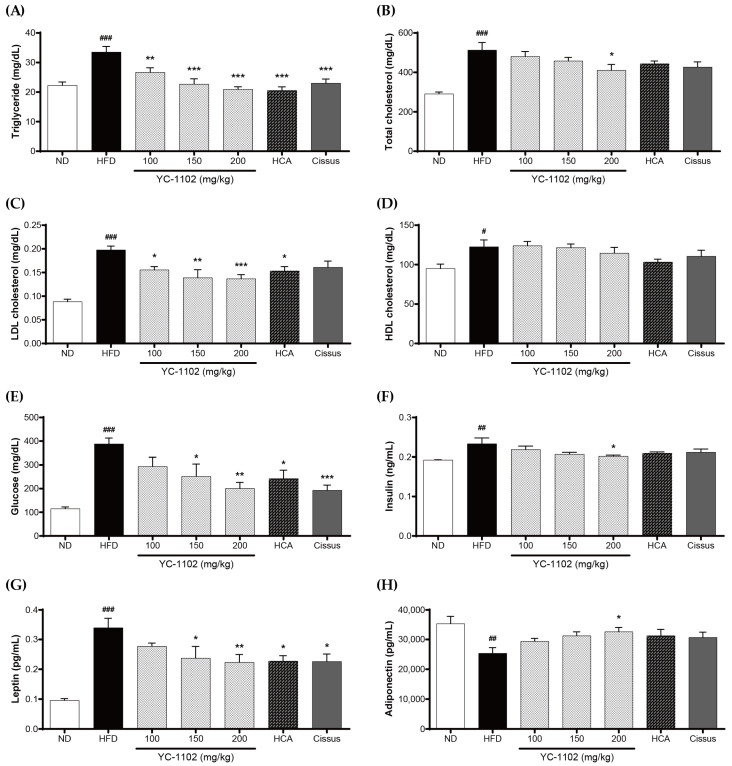
Effect of YC-1102, HCA, and Cissus on serum profiles in HFD-fed mice. (**A**) Triglyceride, (**B**) total cholesterol, (**C**) LDL cholesterol, (**D**) HDL cholesterol, (**E**) glucose, (**F**) insulin, (**G**) leptin, and (**H**) adiponectin. Values are expressed as the mean ± SD (ND group: n = 7 mice/group; other groups: n = 8 mice/group). # *p* < 0.05, ## *p* < 0.01, and ### *p* < 0.001 vs. ND group, * *p* < 0.05, ** *p* < 0.01, and *** *p* < 0.001 vs. HFD group.

The effects of YC-1102 on the lipid parameters (TG, TC, and LDL cholesterol and HDL cholesterol), glucose, and hormone (insulin, leptin, and adiponectin) levels in the test animals were determined. The levels of all lipid parameters (TG, TC, and LDL cholesterol and HDL cholesterol) were increased by the HFD, whereas the YC-1102 groups exhibited a reduction in the plasma levels of TG, TC, and LDL cholesterol (Figure 4A–C); however, the level of HDL cholesterol did not change (Figure 4D). Compared to the ND group, the HFD group showed increased levels of glucose, insulin, and leptin as well as a decrease in adiponectin, whereas the YC-1102 group showed a significant reduction at the highest dose in the levels of glucose, insulin, and leptin and restored adiponectin levels (Figure 4E–H).

### 3.5. Effects of YC-1102 on eWAT Size and Gene Expressions Related to Adipogenesis and Thermogenesis

**Figure 5:**
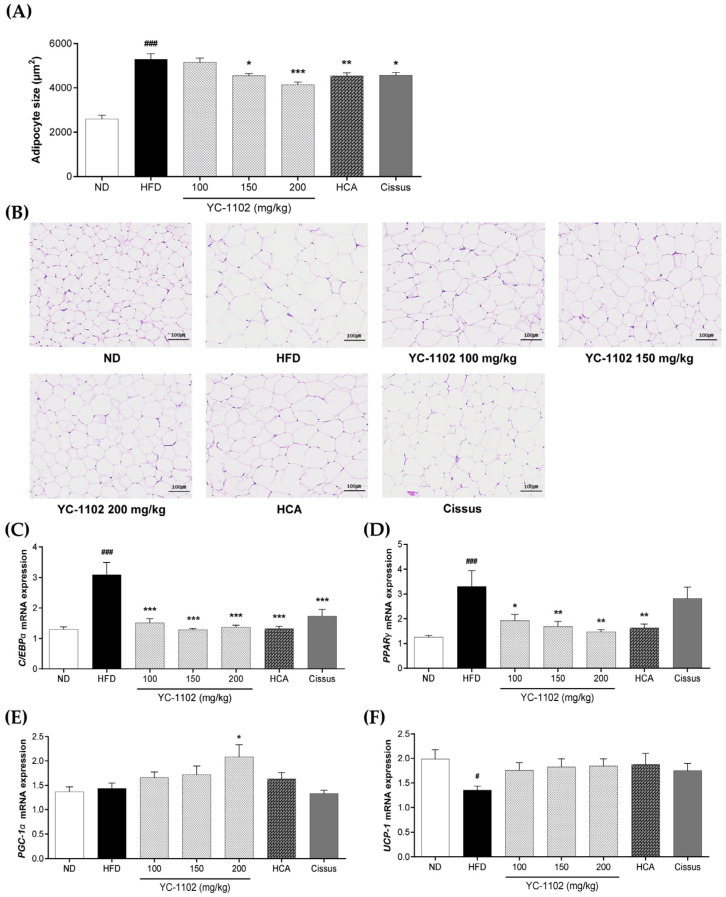
Effect of YC-1102 on adipose tissue in HFD-fed mice. (**A**) Adipocyte tissue size. (**B**) Histology of adipose tissue. Levels of (**C**) *C/ERPα*, (**D**) *PPARγ*, (**E**) *PGC-1α*, and (**F**) *UCP-1*. Values are expressed as the mean ± SD (ND group: n = 7 mice/group; other groups: n = 8 mice/group). # *p* < 0.05 and ### *p* < 0.001 vs. ND group. * *p* < 0.05, ** *p* < 0.01, and *** *p* < 0.001 vs. HFD group.

The size of adipocytes in the eWAT of test animals increased 2-fold or higher due to the HFD, and the administration of YC-1102 decreased the adipocyte size (Figure 5A,B). To verify the effect of YC-1102 on adipocyte size in eWAT, the mRNA expressions of *PPARγ* and *C/EBPα*, which are factors related to adipogenesis, and of *PGC-1α* and *UCP-1*, which are factors related to thermogenesis, were analyzed using real-time PCR (Figure 5C–F). *PPARγ* and *C/EBPα* expression levels were significantly higher in the HFD group than in the ND group. The YC-1102-treated groups showed a significant decrease in the mRNA expressions of *PPARγ* and *C/EBPα*, similar to the HCA group. The levels of *PGC-1α* involved in thermogenesis tended to increase significantly in the YC-1102-treated groups compared to that in the HFD group. However, treatment with HCA or Cissus did not show a significant effect in the expression of this thermogenesis-related marker. The expression levels of *UCP-1* showed an increase in the YC-1102-treated groups, but no significant difference was observed compared with the other HFD-fed groups.

## 4. Discussion

Obesity has become a major public health issue worldwide, and current statistics indicate that 40% of adults and 20% of children are obese [1]. During cell proliferation, the number of cells remains constant; thus, an increase in adipocyte size is associated with weight control [25]. Obesity is characterized by the excessive proliferation and enlargement of adipose tissue. It is associated with metabolic disorders caused by various inflammatory factors released by the adipose tissues that regulate immune responses [26,27].

Cell proliferation in adipose tissues involves the generation and enlargement of adipocytes; this process is regulated via the interactions between the transcription factors PPARγ and C/EBPα [26]. Additionally, the transcription factor SREBP-1c contributes to PPARγ expression and partially regulates C/EBPα during adipogenesis [28]. In this study, YC-1102, an extract from the roots of *R. multiflora*, was shown to inhibit the generation of TGs in adipocytes differentiated from 3T3-L1 cells and effectively reduce body weight, body fat, organ weight, and adipocyte size in HFD-induced obese mice. YC-1102 was also shown to significantly inhibit the mRNA and protein expressions of PPARγ and C/EBPα both in 3T3-L1 adipocytes and the eWAT of HFD-induced obese mice. In particular, the experimental groups administered medium (150 mg/kg) and high (200 mg/kg) doses of YC-1102 showed better reduction effects in C/EBPα and PPARγ mRNA expressions than the Cissus-treated group and comparable to that of the HCA-treated group. These results suggest that YC-1102 exerts potent anti-obesity effects by inhibiting adipocyte differentiation and proliferation.

In addition to the regulation of lipid absorption and decomposition, obesity is also regulated by energy metabolism, which alters mitochondrial biogenesis, fatty acid oxidation, and thermogenesis [29,30,31]. Obesity leads to mitochondrial dysfunction, adipokine secretion, and increased fat accumulation [32]. Consequently, it can reduce body fat by enhancing energy metabolism via the regulation of mitochondrial biogenesis and fatty acid oxidation [29,33]. PGC-1α is a transcriptional coactivator that controls the expression of numerous genes, including PPAR, Forkhead box O (FOXO), and SREBP [34]. It is also a master regulator of mitochondrial biogenesis because it facilitates fatty acid transport and utilization [34,35]. In contrast, UCP-1 induces energy consumption via thermogenesis based on the potential difference between the outer and inner mitochondrial membranes [36]. Thermogensis is controlled by pathways, including beta-adrenergic receptor-cAMP, mitogen-activated protein kinase, and AMP-activated protein kinase, resulting in the production of fat by means of an increase in energy expenditure and thermogenesis via the regulation of mitochondrial activity [37,38]. In this study, YC-1102 significantly increased the mRNA and protein expressions of PGC-1α in a dose-dependent manner, both in adipocytes differentiated from 3T3-L1 cells and in the eWAT of HFD-induced obese mice. In addition, PGC-1α expression in the 200 mg/kg YC-1102-treated group was to a greater extent than HCA or Cissus, so that it can be expected that YC-1102 can improve thermogenesis. Although the expression levels of UCP-1 showed an increase in the YC-1102-treated groups, no significant difference was observed compared with the other HFD-fed groups. The epididymal fat belongs to visceral fat tissue. The perigonadal fat pads are typically the largest and most readily accessible fat pads and, for these and other reasons, they are the most frequently used in the numerous studies [39,40,41]. Lipids are mainly stored in white adipocytes [42]. Differentiated 3T3-L1 adipocytes are a widely used in vitro model of white adipocytes [43]. Thus, in the present study, the PGC-1α/UCP-1 pathway was evaluated in 3T3-L1 cells and eWAT. Although, the expression of UCP-1 is mainly found in brown adipose tissue, the possibility of heat production through the PGC-1α/UCP-1 pathway could also be evaluated in WAT [44]. These results demonstrate that the regulation of thermogenesis by using YC-1102 treatment can prevent WAT proliferation by increasing fatty acid oxidation and energy consumption.

Adipose tissues secrete substances with a regulatory role in metabolism, in addition to their function in the storage of TG [37]. These substances, including leptin and adiponectin, are collectively referred to as adipokines, which regulate obesity and cardiovascular diseases [37]. Leptin is produced in proportion to body fat as the main protein in adipose tissue and is a hormone involved in energy homeostasis and neuroendocrine functions [38]. The concentration of circulating leptin in the blood is high in obese patients, and chronic leptin secretion caused by obesity can induce leptin resistance [45]. Adiponectin is mostly expressed in adipose tissue, and plasma adiponectin concentrations decrease in patients with obesity or cardiovascular disease [46,47]. Studies have shown that adiponectin increases insulin sensitivity and stimulates fatty acid oxidation by preventing lipid accumulation in skeletal muscles and the liver. Adiponectin has also been reported to exert anti-inflammatory effects [48]. In HFD-induced obese mice, the level of leptin increased, and the level of adiponectin decreased; the administration of YC-1102 decreased leptin levels and increased adiponectin levels in a dose-dependent manner, implying an ameliorating effect of YC-1102 on obesity and its related symptoms.

YC-1102 contains various components such as rosamultin, euscaphic acid, and β-sitosterol. In a previous study, rosamultin reduced triglyceride levels in 3T3-L1 cells [20]. Euscaphic acid improved a HFD-induced hyperlipidemia in rats [49]. β-sitosterol decreased hepatic lipid accumulation in mice fed a high-fat Western-style diet [50]. Although the detailed mechanism of YC-1102 as an anti-obesity herbal supplement is still not clear, it is suggested that the anti-obesity activity of YC-1102 may be due to the synergistic effects of these compounds.

## 5. Conclusions

Our findings show that YC-1102 has a positive effect on the regulation of adipogenesis and energy metabolism in 3T3-L1 preadipocytes and in HFD-induced obese mice. YC-1102 was found to downregulate the expressions of PPARγ and C/EBPα during adipogenesis, inhibiting adipocyte differentiation and upregulating the expression of PGC-1α for energy metabolism to increase mitochondrial biogenesis and fatty acid oxidation. Daily administration of YC-1102 to HFD-fed mice prevented increases in body weight and the accumulation of body fat in a dose-dependent manner. YC-1102 administration also led to a decrease in TG, TC, and LDL cholesterol levels, as well as glucose and leptin levels, and an increase in adiponectin levels, thereby effectively inhibiting lipid metabolism. Based on these results, we conclude that YC-1102 has a dual mechanism that reduces the expression of transcription factors that promote the adipocyte differentiation and increases the expression of factors that promote energy consumption. Several toxicity studies were conducted to confirm its safety profiles before clinical tests in humans. YC-1102 was tested in rats (per oral) for GLP toxicity studies such as single-dose toxicity (lethal dose > 5000 mg/kg), 28-day repeat-dose toxicity (NOAEL 5000 mg/kg/day), and 90-day repeat-dose toxicity (NOAEL 5000 mg/kg/day). YC-1102 could be used as a potential ingredient in health supplement foods for daily administration to prevent lifestyle-induced obesity by weight loss and lipid profile improvement.

## Figures and Tables

**Table 1:** List of primers used for PCR.

Gene	Primer	Sequences (5′ → 3′)
C/EBPα	Forward	GCGGGAACGCAACAACATC
	Reverse	GTCACTGGTCAACTCCAGCAC
PPARγ	Forward	TTCGCTGATGCACTGCCTATGA
	Reverse	AAGGAATGCGAGTGGTCTTCCA
PGC-1α	Forward	TGTTCCCGATCACCATATTCC
	Reverse	GGTGTCTGTAGTGGCTTGATTC
UCP-1	Forward	GTGAACCCGACAACTTCCGAA
	Reverse	TGAAACTCCGGCTGAGAAGAT
GAPDH	Forward	AGGTCTGGTGTGAACGGATTTG
	Reverse	TGTAGACCATGTAGTTGAGGTCA

## Data Availability

The datasets generated and/or analyzed during the current study are not publicly available due to confidentiality but are available from the corresponding author on reasonable request.

## References

[1] Lin X., Li H. (2021). Obesity: Epidemiology, Pathophysiology, and Therapeutics. Front. Endocrinol..

[2] Longo M., Zatterale F., Naderi J., Parrillo L., Formisano P., Raciti G.A., Beguinot F., Miele C. (2019). Adipose Tissue Dysfunction as Determinant of Obesity-Associated Metabolic Complications. Int. J. Mol. Sci..

[3] Tang Q.Q., Otto T.C., Lane M.D. (2003). Mitotic clonal expansion: A synchronous process required for adipogenesis. Proc. Natl. Acad. Sci. USA.

[4] Yudkin J.S. (2007). Inflammation, obesity, and the metabolic syndrome. Horm. Metab. Res..

[5] Calle E.E., Rodriguez C., Walker-Thurmond K., Thun M.J. (2003). Overweight, obesity, and mortality from cancer in a prospectively studied cohort of U.S. adults. N. Engl. J. Med..

[6] Botchlett R., Woo S.L., Liu M., Pei Y., Guo X., Li H., Wu C. (2017). Nutritional approaches for managing obesity-associated metabolic diseases. J. Endocrinol..

[7] Choi D.H., Han J.H., Yu K.H., Hong M., Lee S.Y., Park K.H., Lee S.U., Kwon T.H. (2020). Antioxidant and Anti-Obesity Activities of Polygonum cuspidatum Extract through Alleviation of Lipid Accumulation on 3T3-L1 Adipocytes. J. Microbiol. Biotechnol..

[8] Moseti D., Regassa A., Kim W.K. (2016). Molecular Regulation of Adipogenesis and Potential Anti-Adipogenic Bioactive Molecules. Int. J. Mol. Sci..

[9] Hong F., Pan S., Guo Y., Xu P., Zhai Y. (2019). PPARs as Nuclear Receptors for Nutrient and Energy Metabolism. Molecules.

[10] Giralt M., Villarroya F. (2013). White, brown, beige/brite: Different adipose cells for different functions?. Endocrinology.

[11] Cheng C.F., Ku H.C., Lin H. (2018). PGC-1alpha as a Pivotal Factor in Lipid and Metabolic Regulation. Int. J. Mol. Sci..

[12] Kim H.L., Park J., Jung Y., Ahn K.S., Um J.Y. (2019). Platycodin D, a novel activator of AMP-activated protein kinase, attenuates obesity in db/db mice via regulation of adipogenesis and thermogenesis. Phytomedicine.

[13] Qiang L., Wang L., Kon N., Zhao W., Lee S., Zhang Y., Rosenbaum M., Zhao Y., Gu W., Farmer S.R. (2012). Brown remodeling of white adipose tissue by SirT1-dependent deacetylation of Ppargamma. Cell.

[14] Ali A.T., Hochfeld W.E., Myburgh R., Pepper M.S. (2013). Adipocyte and adipogenesis. Eur. J. Cell Biol..

[15] Ghaben A.L., Scherer P.E. (2019). Adipogenesis and metabolic health. Nat. Rev. Mol. Cell Biol..

[16] Van Gaal L., Pi-Sunyer X., Despres J.P., McCarthy C., Scheen A. (2008). Efficacy and safety of rimonabant for improvement of multiple cardiometabolic risk factors in overweight/obese patients: Pooled 1-year data from the Rimonabant in Obesity (RIO) program. Diabetes Care.

[17] Pi-Sunyer X., Astrup A., Fujioka K., Greenway F., Halpern A., Krempf M., Lau D.C., le Roux C.W., Violante Ortiz R., Jensen C.B. (2015). A randomized, controlled trial of 3.0 mg of liraglutide in weight management. N. Engl. J. Med..

[18] Li Y.L. (2017). A new triterpenic acid from roots of Rosa multiflora var. cathayensis. Chin. Tradit. Herb. Drugs.

[19] Park K.H., Kim S.K., Choi S.E., Kwon J.H., Oh M.H., Lee M.W. (2010). Three new stereoisomers of condensed tannins from the roots of Rosa multiflora. Chem. Pharm. Bull..

[20] Lin S., Zhao X., Sun Y., Liu H., Shang M., Gong J., Ma Q., Piao G., Yuan H. (2020). Inhibitory effects of compounds from the roots of Potentilla longifolia on lipid accumulation. PLoS ONE.

[21] Cheol Park J., Chul Kim S., Moon Hur J., Choi S.H., Yeon Lee K., Won Choi J. (2004). Anti-hepatotoxic effects of Rosa rugosa root and its compound, rosamultin, in rats intoxicated with bromobenzene. J. Med. Food.

[22] Choi J.H., Baek J.Y., Choi H.J. (2015). Effects of Rosa multiflora and Rosa multiflora complex on lipid content in rats fed a high-fat high-cholesterol diet. J. Korean Soc. Food Sci. Nutr..

[23] Park K.H., Choi S.E., Choi Y.W., Lee D.I., Joo S.S., Jeong M.S., Bang H., Lee C.S., Lee M.K., Seo S.J. (2011). Topical application of two condensed tannins from the root of Rosa multiflora Thunberg for the treatment of atopic dermatitis (AD) in NC/Nga mice. Phytother. Res..

[24] Semwal R.B., Semwal D.K., Vermaak I., Viljoen A. (2015). A comprehensive scientific overview of Garcinia cambogia. Fitoterapia.

[25] Spalding K.L., Arner E., Westermark P.O., Bernard S., Buchholz B.A., Bergmann O., Blomqvist L., Hoffstedt J., Naslund E., Britton T. (2008). Dynamics of fat cell turnover in humans. Nature.

[26] Kusminski C.M., Bickel P.E., Scherer P.E. (2016). Targeting adipose tissue in the treatment of obesity-associated diabetes. Nat. Rev. Drug Discov..

[27] Ibrahim M.M. (2010). Subcutaneous and visceral adipose tissue: Structural and functional differences. Obes. Rev..

[28] Payne V.A., Au W.S., Lowe C.E., Rahman S.M., Friedman J.E., O’Rahilly S., Rochford J.J. (2009). C/EBP transcription factors regulate SREBP1c gene expression during adipogenesis. Biochem. J..

[29] Lee J.H., Park A., Oh K.J., Lee S.C., Kim W.K., Bae K.H. (2019). The role of adipose tissue mitochondria: Regulation of mitochondrial function for the treatment of metabolic diseases. Int. J. Mol. Sci..

[30] Ye Z., Wang S., Zhang C., Zhao Y. (2020). Coordinated modulation of energy metabolism and inflammation by branched-chain amino acids and fatty acids. Front. Endocrinol..

[31] Pan R., Zhu X., Maretich P., Chen Y. (2020). Combating obesity with thermogenic fat: Current challenges and advancements. Front. Endocrinol..

[32] de Mello A.H., Costa A.B., Engel J.D.G., Rezin G.T. (2018). Mitochondrial dysfunction in obesity. Life Sci..

[33] Serra D., Mera P., Malandrino M.I., Mir J.F., Herrero L. (2013). Mitochondrial fatty acid oxidation in obesity. Antioxid. Redox Signal..

[34] Fernandez-Marcos P.J., Auwerx J. (2011). Regulation of PGC-1alpha, a nodal regulator of mitochondrial biogenesis. Am. J. Clin. Nutr..

[35] Jornayvaz F.R., Shulman G.I. (2010). Regulation of mitochondrial biogenesis. Essays Biochem..

[36] Busiello R.A., Savarese S., Lombardi A. (2015). Mitochondrial uncoupling proteins and energy metabolism. Front. Physiol..

[37] Lancha A., Fruhbeck G., Gomez-Ambrosi J. (2012). Peripheral signalling involved in energy homeostasis control. Nutr. Res. Rev..

[38] Bluher M., Mantzoros C.S. (2015). From leptin to other adipokines in health and disease: Facts and expectations at the beginning of the 21st century. Metabolism.

[39] Neuhofer A., Zeyda M., Mascher D., Itariu B.K., Murano I., Leitner L., Hochbrugger E.E., Fraisl P., Cinti S., Serhan C.N. (2013). Impaired local production of proresolving lipid mediators in obesity and 17-HDHA as a potential treatment for obesity-associated inflammation. Diabetes.

[40] Bluher M., Michael M.D., Peroni O.D., Ueki K., Carter N., Kahn B.B., Kahn C.R. (2002). Adipose tissue selective insulin receptor knockout protects against obesity and obesity-related glucose intolerance. Dev. Cell.

[41] Chang Y.C., Yu Y.H., Shew J.Y., Lee W.J., Hwang J.J., Chen Y.H., Chen Y.R., Wei P.C., Chuang L.M., Lee W.H. (2013). Deficiency of NPGPx, an oxidative stress sensor, leads to obesity in mice and human. EMBO Mol. Med..

[42] Spalding K.L., Bernard S., Naslund E., Salehpour M., Possnert G., Appelsved L., Fu K.Y., Alkass K., Druid H., Thorell A. (2017). Impact of fat mass and distribution on lipid turnover in human adipose tissue. Nat. Commun..

[43] Morrison S., McGee S.L. (2015). 3T3-L1 adipocytes display phenotypic characteristics of multiple adipocyte lineages. Adipocyte.

[44] Ringholm S., Grunnet Knudsen J., Leick L., Lundgaard A., Munk Nielsen M., Pilegaard H. (2013). PGC-1alpha is required for exercise- and exercise training-induced UCP1 up-regulation in mouse white adipose tissue. PLoS ONE.

[45] Myers M.G., Cowley M.A., Munzberg H. (2008). Mechanisms of leptin action and leptin resistance. Annu. Rev. Physiol..

[46] Scherer P.E., Williams S., Fogliano M., Baldini G., Lodish H.F. (1995). A novel serum protein similar to C1q, produced exclusively in adipocytes. J. Biol. Chem..

[47] Scheid M.P., Sweeney G. (2014). The role of adiponectin signaling in metabolic syndrome and cancer. Rev. Endocr. Metab. Disord..

[48] Kadowaki T., Yamauchi T., Kubota N., Hara K., Ueki K., Tobe K. (2006). Adiponectin and adiponectin receptors in insulin resistance, diabetes, and the metabolic syndrome. J. Clin. Investig..

[49] Park H.J., Nam J.H., Jung H.J., Lee M.S., Lee K.T., Jung M.H., Choi J. (2005). Inhibitory effect of euscaphic acid and and tormentic acid from the roots of Rosa rugosa on high fat diet-induced obesity in the rat. Korean J. Pharmacogn..

[50] Feng S., Dai Z., Liu A.B., Huang J., Narsipur N., Guo G., Kong B., Reuhl K., Lu W., Luo Z. (2018). Intake of stigmasterol and beta-sitosterol alters lipid metabolism and alleviates NAFLD in mice fed a high-fat western-style diet. Biochim. Biophys. Acta Mol. Cell Biol. Lipids.

